# A Magnetic-Assisted CRISPR-Cas12a Biosensor Incorporating a Y-DNA Probe for Sensitive Detection of *Schistosoma japonicum* Eggs

**DOI:** 10.3390/bios16050293

**Published:** 2026-05-18

**Authors:** Ting Liu, Haogang Guo, Mengmeng Yu, Jiawei Peng, Liwen Guan, Shuying Xie, Xian Hao, Yifei Yang

**Affiliations:** 1 Jiangxi Provincial Key Laboratory of Preventive Medicine, School of Public Health, Nanchang University, Nanchang 330031, China; liutingo20@163.com (T.L.); yumengmengjx@163.com (M.Y.); 13627968672@163.com (J.P.); guanliwen116@163.com (L.G.); 2 School of Queen Mary, Nanchang University, Nanchang 330031, China; guohaogang2004@163.com; 3 Jiangxi Provincial Institute of Parasitic Diseases, Nanchang 330031, China; xsy0317@163.com

**Keywords:** *Schistosoma japonicum* egg, CRISPR-Cas12a, aptamer, magnetic separation, DNA structure

## Abstract

*Schistosomiasis*, caused by Schistosoma species, is notoriously difficult to accurately diagnose with conventional methods. In this study, we present an innovative biosensor that integrates CRISPR–Cas12a technology with nucleic acid aptamers for the highly sensitive detection of *Schistosoma japonicum* eggs. The biosensor leverages a Y-shaped DNA structure (Y-DNA) that incorporates an aptamer specific to *S. japonicum* eggs, along with an activator DNA and a segment for immobilization on magnetic nanomaterials. Upon target recognition, the Y-DNA releases the activator, which triggers the collateral cleavage activity of Cas12a, enabling the direct detection of eggs. This system demonstrates remarkable sensitivity, being capable of detecting individual eggs in infected rabbit serum and feces. Moreover, it effectively distinguishes the eggs of *S. japonicum* from those of other parasitic species. The simplicity, high sensitivity, and rapid detection of our biosensor offer significant potential for improving the diagnosis of schistosomiasis, providing a novel, reliable tool for early detection in clinical settings.

## 1. Introduction

*Schistosomiasis* is a zoonotic parasitic disease caused by *Schistosoma* species, affecting approximately 250 million people worldwide and ranking second only to malaria in global impact [1]. Three primary species—*Schistosoma japonicum*, *Schistosoma mansoni*, and *Schistosoma haematobium*—cause human infections, with *S. japonicum* primarily distributed in regions of China, the Philippines, and Indonesia [2]. While schistosomiasis has been largely controlled in many areas through the widespread use of anthelmintic treatments, including praziquantel, the rise of asymptomatic carriers with low worm burdens presents significant challenges for accurate diagnosis and eradication efforts [3,4]. For these asymptomatic individuals, who show few or no symptoms in the early stages of infection, highly sensitive diagnostic tools are required to ensure timely intervention and effective control strategies.

*Schistosomiasis* detection methods include traditional parasitology, immunological diagnosis, and nucleic acid detection. The Kato–Katz (K-K) technique, based on microscopic examination of fecal eggs, remains the gold standard for diagnosing *schistosomiasis* due to its cost-effectiveness, convenience, and specificity. However, its sensitivity is reduced in cases of low-intensity infections [5]. The miracidium hatching technique (MHT) is more sensitive but time-consuming [6]. Immunological methods are widely used for preliminary screening and disease surveillance in endemic regions due to their low cost, ease of use, and sensitivity [7]. However, these methods may cross-react with other parasitic diseases and cannot distinguish between past and current infections [8]. Nucleic acid detection, which has high sensitivity and specificity, offers new diagnostic potential and can be applied to various sample types, including environmental samples, serum, and feces [9,10,11]. However, it requires expensive equipment and specialized professionals for DNA extraction, limiting its accessibility. Given the need for more sensitive, specific, and simpler diagnostic methods, rapid identification of *schistosomiasis* eggs is crucial for effective control and clinical management.

Recent advancements have sought to improve K-K detection, such as the Helmintex egg enrichment system [12] and egg capture microfiltration devices [13], which enhance microscopic sensitivity. Additionally, webcam-assisted real-time observation using algorithms to count eggs [14] has been introduced. While these improvements boost sensitivity, they still rely on optical microscopy and require professional expertise, and they cannot overcome the challenges of low specificity and time consumption. Aptamers, which can specifically bind to *S. japonicum* eggs, have been developed using the SELEX technique [15]. These aptamers offer high binding affinity, specificity, a low molecular weight, stability, ease of synthesis and modification, and non-immunogenicity, making them ideal for biosensor development [16,17]. The CRISPR-Cas system, consisting of clustered regularly interspaced short palindromic repeats (CRISPRs) and CRISPR-associated proteins (Cas), has emerged as a novel biosensing tool, compatible with various biotechnologies [18,19,20,21,22]. CRISPR-Cas12a (Cpf1), known for its strong specificity, high sensitivity, and low cost, has become the most widely used technology in biosensors for applications ranging from food and drug analysis to clinical diagnosis and environmental detection [23,24,25,26]. The combination of aptamer-based functional nucleic acids [27] and bacterial allosteric transcription factors [28] further extends the utility of CRISPR-Cas12a, enabling the detection of non-nucleic acid targets and broadening its potential for in vitro diagnostics.

This study introduces a novel approach by combining aptamer-based recognition of *S. japonicum* eggs with the CRISPR-Cas12a biosensing system. We developed a magnetic-assisted Y-DNA probe-regulated CRISPR-Cas12a biosensor, utilizing gold-coated magnetic nanoparticles (AuMNPs) to facilitate the detection process. The Y-DNA probe, immobilized on the surface of AuMNPs, captures *S. japonicum* eggs, triggering Cas12a cleavage activity and producing a fluorescence signal for rapid, sensitive detection. Notably, this system detects *S. japonicum* eggs without the need for DNA extraction, making it suitable for use with various sample types, including serum and feces, in clinical settings. By offering a highly sensitive, cost-effective, and user-friendly detection method, this innovative biosensor has the potential to significantly improve the early diagnosis and surveillance of *schistosomiasis*, contributing to the global effort to control and ultimately eradicate this neglected tropical disease.

## 2. Materials and Methods

The details of all materials and reagents, gel electrophoresis and fluorescence measurement, oligonucleotides (Table S1), synthesis of gold nanoparticles (AuNPs), synthesis of Fe_3_O_4_, and preparation of AuNPs labeling Fe_3_O_4_ nanoparticles (AuMNPs) are described in the Supporting Information.

### 2.1. Modification of Y-Shaped DNA Probe (Y-DNA) on AuMNPs

To avoid the non-specific adsorption of ssDNA on the surface of AuMNPs, excessive thioated fDNA was added to occupy the binding site [29], thus allowing the Y-DNA probe to be modified on AuMNPs (AuMNPs@Y-DNA). In short, 150 µL 10 µM thioated fDNA was incubated with 15 µL 10 mM TCEP at room temperature for 1 h to activate mercaptan groups. Then, 1.5 mL of AuMNP supernatant obtained after magnetic separation was dispersed in 600 µL PBS (10 mM, pH = 7.4). Finally, 150 µL TCEP-treated fDNA was added to the AuMNP solution and reacted overnight at 4 °C. After the completion of the reaction, the unbound fDNA was removed by magnetic separation and washed with ultra-pure water 3 times. Then, AuMNPs@fDNA particles were dispersed in 500 µL PBS solution containing 2 µM cDNA and 2 µM activator and incubated at room temperature for 2 h. At the end of the reaction, the supernatant obtained after magnetic separation was discarded, and then the remaining sample was washed with ultra-pure water 3 times. Finally, AuMNPs@Y-DNA was added to 1.5 mL PBS solution and stored at 4 °C. Subsequently, 150 µL of TCEP-treated fDNA was added to the AuMNP solution and reacted overnight at 4 °C. Then, unbound fDNA was removed through magnetic separation and washed three times with ultrapure water. The AuMNPs@fDNA particles were dispersed in 500 μL of PBS solution containing 2 µM cDNA and 2 µM activator, and then they were incubated at room temperature for 2 h. After the reaction, the supernatant obtained after the magnetic separation was discarded, and the particles were washed three times with ultrapure water. Finally, the AuMNPs@Y-DNA was added to 1.5 mL of PBS solution and stored at 4 °C until use.

### 2.2. Detection of Schistosoma Japonicum Eggs

The detection system that we constructed comprises two components: a reaction solution and a report solution. The reaction solution was prepared by first magnetically separating 100 µL of AuMNPs@Y-DNA and discarding the supernatant. Next, 20 µL of sample solution (containing NEBuffer r2.1) was added, and the supernatant was obtained after magnetic separation following a 1 h incubation. The report solution was prepared as follows: A 10 µL mixture of Cas12a-crRNA, comprising 1.25 µL of 1 µM crRNA, 2.5 µL of 1 µM LbaCas12a, 1 µL of 10× NEBuffer r2.1, and 5.25 µL of H_2_O, was briefly swirled on an oscillator and incubated at 37 °C to form a Cas12a-crRNA complex. Then, 5 µL of a 2 µM ssDNA-FQ solution (containing NEBuffer r2.1) was added, resulting in the formation of the report solution. Subsequently, 10 µL of the reaction solution was combined with 15 µL of the report solution, followed by incubation at 37 °C, and fluorescence signals were collected using either a fluorescence spectrophotometer or real-time PCR. Fluorescence spectrophotometry was utilized to measure the fluorescence levels after 60 min of incubation at 37 °C and 10 min at 65 °C, with an excitation wavelength of 485 nm and an emission wavelength of 520 nm. To investigate the detection potential of the magnetic-assisted CRISPR-Cas12a biosensor detection platform regulated by Y-DNA for biological samples, we tested feces obtained from rabbits in the advanced stages of *S. japonicum* infection, as well as uninfected rabbit feces. The rabbit feces were dissolved in water, and an appropriate amount (200 nm) of Fe_3_O_4_ was added for the magnetic adsorption of worm eggs. The mixture was shaken and mixed for 5 min, followed by magnetic separation and three rounds of washing. This process yielded relatively clean eggs. Finally, the magnetically separated Fe_3_O_4_ was introduced into the detection system to measure the fluorescence intensity of the solution.

### 2.3. Statistical Analysis

All experiments were performed with at least three independent replicates. Data are presented as the mean ± standard deviation (SD). Graphical representations and statistical analyses were performed using GraphPad Prism 8.0. Comparisons between two groups were conducted using the *t*-test, whereas comparisons among multiple groups were performed using a one-way analysis of variance (one-way ANOVA). Differences were considered statistically significant at *p* < 0.05 (* *p* < 0.05, ** *p* < 0.01, *** *p* < 0.001, and **** *p* < 0.0001).

## 3. Results and Discussion

### 3.1. Principle of the Method

The magnetic-assisted Y-DNA-regulated CRISPR-Cas12a sensor that we constructed consists of two parts: a probe system and a reporting system. The AuMNPs@Y-DNA nanoprobe constitutes the probe system, wherein Y-DNA is composed of fixed DNA (fDNA), activator DNA (activator), and capture DNA (cDNA), which form a stable Y-shaped structure through complementary base pairing. The fDNA is attached to the surface of AuMNPs by S-Au bonds, while the activator regulates the CRISPR-Cas12a biosensor, and cDNA serves as the nucleic acid aptamer that specifically binds to *S. japonicum* eggs. The synthesis process of the AuMNPs@Y-DNA nanoprobes: monodisperse Fe_3_O_4_ nanoparticles are synthesized using a solvothermal reaction, followed by modification with polyethyleneimine and gold nanoparticles on the surface of Fe_3_O_4_ via ultrasound, yielding magnetic AuMNPs. Finally, the Y-DNA probe is immobilized on the surface of the AuMNPs to obtain the AuMNPs@Y-DNA nanoprobe. The reporting system comprises pre-assembled Cas12a-crRNA complexes and single-stranded DNA fluorescence reporter molecules (ssDNA-FQ) labeled with fluorophores (FAM) and quenching agents (BHQ1) at both ends. In the absence of eggs, Y-DNA is stably connected to AuMNPs, locking the activator, which prevents the binding of the activator to the Cas12a-crRNA complex. Therefore, the DNase activity of Cas12a cannot be activated, the ssDNA-FQ fluorescence reporter molecule remains intact, and the fluorescence signal remains almost unchanged. When the egg target is present, the cDNA on the Y-DNA probe recognizes and captures the egg, causing the cDNA to fall off from the Y-DNA probe system, resulting in the unstable connection of the activator to the probe and shedding. After magnetic separation, the supernatant containing the activator is added to the reporting system. The activator binds with Cas12a-crRNA to form a ternary complex and activates the trans-cleavage activity of Cas12a to cut ssDNA-FQ fluorescence signal molecules. As a result, the fluorophore is separated from the quenching agent, resulting in a significant fluorescence signal that enables sensitive and specific detection of *S. japonicum* eggs in the sample.

### 3.2. Characterization of Y-DNA on AuMNPs

In order to connect Y-DNA to the surface of magnetic nanoparticles, we chose AuNPs as the connection bridge. On the one hand, AuNPs can be coated on the surface of Fe_3_O_4_ particles under the action of PEI. On the other hand, the Y-DNA probe can be connected to AuNPs by S-Au bonds. Colloidal AuNPs with a particle size of about 2~3 nm were prepared by reducing chloroauric acid with sodium borohydride [30]. Scanning electron microscope (SEM) and atomic force microscope (AFM) images showed that the AuNPs were spherical and that the particles were very small (Figure S1A,B).

**Figure 1:**
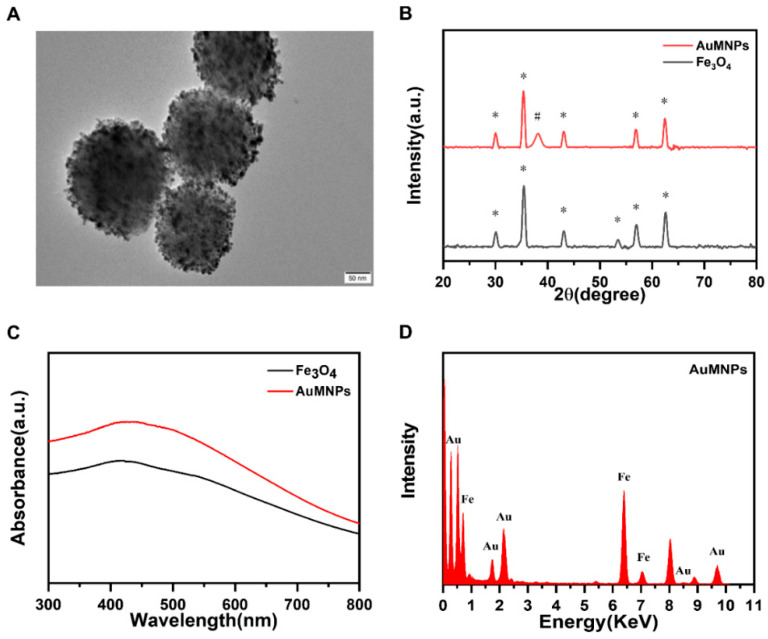
(**A**) TEM image of AuMNPs. (**B**) XRD pattern of AuMNPs (* represents Fe_3_O_4_ characteristic diffraction peak, # represents Au characteristic diffraction peak). (**C**) UV–vis spectrum of Fe_3_O_4_ and AuMNPs. (**D**) EDS spectrum of AuMNPs.

We synthesized high-performance AuMNP microspheres in three steps. Firstly, Fe_3_O_4_ particles with magnetic properties and a particle size of about 200 nm were synthesized by an improved solvothermal reaction. Secondly, under the conditions of ultrasound, PEI self-assembled onto the surface of Fe_3_O_4_ particles, thereby producing Fe_3_O_4_@PEI microspheres. Finally, monodisperse AuMNP microspheres were obtained by mixing the Fe_3_O_4_@PEI microspheres with AuNPs. The size and morphology of the AuMNPs were characterized by a transmission electron microscope (TEM). As shown in Figure 1A, the diameter of the AuMNP magnetic particles was about 200 nm, showing good dispersion, and there was a large amount of AuNPs on the surface of Fe_3_O_4_. These results show that AuMNP microspheres were successfully prepared with uniform size and good dispersion.

As shown in Figure 1B, the prepared AuMNPs were characterized by a X-ray diffractometer. The black curve shows the X-ray diffraction spectrum of typical Fe_3_O_4_ particles. The diffraction peaks correspond to the (220, 311, 400, 422, 511, 440) faces of cubic inverse spinel Fe_3_O_4_ (JCPDS number: 19-0629), indicating that the obtained products were highly pure Fe_3_O_4_ nanoparticles. After the adsorption of the AuNPs on the Fe_3_O_4_@PEI microspheres, a new X-ray diffraction peak (red curve) appeared, corresponding to the crystal plane of Au (JCPD number: 04-0784). No diffraction peak corresponding to PEI was observed because the PEI layer was amorphous. These results are consistent with those reported by other research teams [31].

Ultraviolet–visible absorption spectra (UV–Vis) of Fe_3_O_4_ and the AuMNP microspheres are shown in Figure 1C. The black curve shows the typical UV–vis spectrum of Fe_3_O_4_. The AuMNP microspheres formed by the coupling of AuNPs and Fe_3_O_4_ do not show an obvious plasmon band (red curve), which indicates that the adsorbed AuNPs were too small to induce plasma coupling. The condition of the Fe_3_O_4_ surface loaded with AuNPs was evaluated by the change in the intensity of AuNP absorption in the supernatant before and after the reaction (Figure S2). The results of the pre-reaction (black curve) and post-reaction (red curve) measurements showed that the absorption intensity of the AuNPs in the supernatant decreased by 40.89% after the reaction, indicating that the AuNPs were efficiently assembled on the surface of Fe_3_O_4_.

To further determine the load of AuNPs on the surface of the AuMNPs, EDS analysis of the AuMNPs was carried out. In the EDS spectrum, we observed the characteristic peaks of Fe and Au, indicating that the AuMNP microspheres contained Fe atoms and Au atoms (Figure 1D). An Fe element mapping point and Au element mapping point can be seen in the Fe and Au element mapping image (Figure S3B,C). The element mapping image shows that the Fe element was mainly located in the core and that the Au element was mainly distributed around it, which is consistent with the TEM result (Figure S3D). To confirm the successful coupling of Y-DNA on the surface of the AuMNPs, we measured the concentration of DNA in the supernatant before and after the reaction to calculate the load of the Y-DNA probe on the surface of the AuMNPs. Figure S4 shows that the absorption intensity of the cDNA and activator declined by 32.02% after the completion of the reaction, confirming that Y-DNA was successfully coupled to the surface of the AuMNPs by Au-S bonds.

### 3.3. The Feasibility of the Y-DNA Probe Structure

To verify the feasibility of detecting *S. japonicum* eggs, Y-DNA must satisfy two requirements: Firstly, it should form a stable Y-shaped structure that effectively locks the activator and prevents it from binding with the Cas12a-crRNA complex, thus reducing background signals. Secondly, when the target is present, cDNA should bind to it and detach from the Y-DNA probe, leading to the destabilization of the activator–probe connection and resulting in a fluorescence signal. In summary, Y-DNA needs to be stable but dissociate when encountering the target.

**Figure 2:**
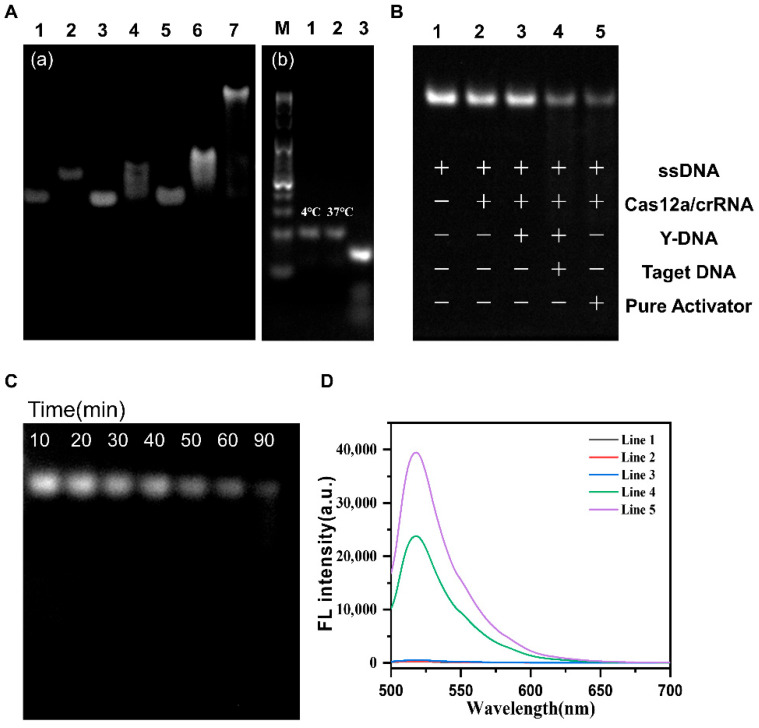
(**A**) Gel analysis of the formation and dissociation of Y-DNA. (**a**) The formation of Y-DNA: lane 1: fDNA; lane 2: cDNA; lane 3: activator; lane 4: fDNA + cDNA; lane 5: fDNA + activator; lane 6: cDNA + activator; lane 7: fDNA + cDNA + activator. (**b**) dissociation of Y-DNA: lane 1: Marker; lane 2: 4 °C Y-DNA; lane 3: 37 °C Y-DNA; lane 4: Y-DNA + tDNA. (**B**) A 12% PAGE gel electrophoresis image of the feasibility of the magnetic-assisted Y-DNA-regulated CRISPR Cas12a biosensor. Lanes 1–5: ssDNA, ssDNA + Cas12a-crRNA, ssDNA + Cas12a-crRNA + Y-DNA, ssDNA + Cas12a-crRNA + Y-DNA + tDNA, ssDNA + Cas12a-crRNA + pure activator. (“+” indicates the presence of the corresponding component, while “−” indicates its absence.) (**C**) A 4% agarose gel electrophoresis image of the trans-cleavage of ssDNA over time. (**D**) Fluorescence spectra of the magnetic-assisted Y-DNA-regulated CRISPR-Cas12a sensor.

We employed polyacrylamide gel electrophoresis (PAGE) to verify the connectivity of the Y-DNA. As shown in the PAGE gel on the left in Figure 2A, lanes 1–3 represent the bands of the simple fDNA (30 nt), cDNA (23 nt), and activator (22 nt), respectively. The double-stranded connection of the three ssDNA pairs indicates that no fDNA–activator complex was formed in lane 5 due to weak affinity between the fDNA and activator (7 base pairs). Bands appeared in both lane 4 and lane 6, representing fDNA–cDNA and cDNA–activator complexes, respectively. Notably, the cDNA–activator band was significantly brighter than the fDNA–cDNA band due to stronger affinity between the cDNA and activator. A new band was observed in lane 7, indicating the successful formation of Y-DNA after simultaneous placement of the fDNA, cDNA, and activator. These results demonstrate the successful construction of a relatively stable Y-DNA probe. Next, we used target DNA (tDNA) complementary to cDNA as a *S. japonicum* egg mimic to react with the Y-DNA probe at 37 °C. The agarose gel on the right in Figure 2A reveals that the bands in lane 2 are consistent with those in lane 1, indicating the stability of the Y-DNA probe at 37 °C. However, upon addition of tDNA, several bands appeared in lane 4, the original Y-DNA bands disappeared, and cDNA-tDNA and two ssDNA bands emerged. The lowest bands corresponded to the fDNA and activator. These results demonstrate that the Y-DNA dissociated and released the activator in the presence of the *S. japonicum* egg mimic (tDNA). In conclusion, we successfully constructed a Y-DNA probe that is both stable and capable of dissociating when encountering the target.

### 3.4. Feasibility of the Magnetic-Assisted Y-DNA-Regulated CRISPR-Cas12a Biosensor

To investigate the regulation and activation of Cas12a-crRNA by the Y-DNA probe, we utilized a 65-base single-stranded DNA (ssDNA) as the CRISPR-Cas12a substrate and confirmed the results using polyacrylamide gel electrophoresis (PAGE), as depicted in Figure 2B. The activator was co-incubated with Cas12a-crRNA, and ssDNA was cleaved, resulting in a lighter band. Conversely, without the activator, ssDNA remained uncleaved, indicating that the activator plays a pivotal role in activating the cleavage activity of Cas12a-crRNA. Additionally, lane 3 revealed that the Y-DNA did not induce DNA cleavage in the absence of the egg mimic (tDNA), proving that the Y-DNA effectively locked the activator and stably connected to the AuMNPs. However, after introducing the egg mimic (tDNA), ssDNA was cleaved, and the band became lighter. Notably, the ssDNA bands gradually faded over time, confirming that Cas12a-crRNA continuously cleaved ssDNA into shorter fragments, which further demonstrated that the egg mimic (tDNA) triggered the efficient cleavage activity of Cas12a-crRNA, as shown in Figure 2C.

To further validate the PAGE analysis, we employed a ssDNA fluorescence reporter (ssDNA-FQ), whose 5′ and 3′ ends were modified with a fluorescence group (FAM) and quenching agent (BHQ1), with only a 5 nt length, to serve as the substrate of Cas12a-crRNA. As illustrated in Figure 2D, adding the activator resulted in an obvious fluorescence signal, which increased with an increasing activator concentration (Figure S5). Moreover, the activator and two other single strands were used to construct Y-DNA probes, which were fixed on AuMNP microspheres. In the absence of the egg mimic, the Y-DNA stably connected to the AuMNPs, and Cas12a could not be activated, resulting in no significant change in the ssDNA-FQ fluorescence signal (Figure 2D, Line 3). However, in the presence of the target, the cDNA on the Y-DNA probe recognized and bound to the egg mimic, causing the activator to fall off from the Y-DNA. The activator then activated Cas12a-crRNA cleavage activity, and the ssDNA-FQ fluorescence signal molecule was cut, producing a significant fluorescence signal (Figure 2D, Line 4). In summary, these results demonstrate that the Y-DNA probes effectively regulate the locking and release of the activator, thereby regulating the cleavage activity of Cas12a-crRNA.

### 3.5. Optimization of Detection Conditions

**Figure 3:**
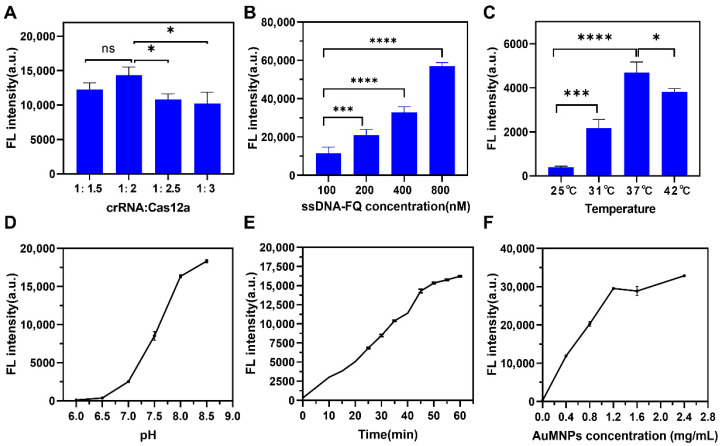
Condition optimization of testing system performance. (**A**) crRNA and Cas12a ratio; (**B**) ssDNA-FQ concentration; (**C**) temperature; (**D**) pH; (**E**) time; (**F**) AuMNP concentration (*n* = 3, * *p* < 0.05, *** *p* <0.001, **** *p* < 0.0001).

In order to achieve optimal detection and analysis performance, we optimized the detection conditions. As depicted in Figure 3A, the fluorescence intensity was the highest when the ratio of crRNA to Cas12a was 1:2; however, as the proportion of Cas12a increased, the fluorescence intensity gradually decreased. Next, we investigated the effect of the ssDNA-FQ concentration on the fluorescence intensity by adding varying concentrations (100–800 nM) to the detection system. The results indicate that the fluorescence intensity increased with an increasing ssDNA-FQ concentration (Figure 3B). This may be attributed to the higher density of ssDNA-FQ around Cas12a at higher concentrations, resulting in more opportunities for Cas12a cleavage and significantly improved efficiency. Subsequently, we explored the effect of the reaction temperature and found that the fluorescence intensity was the highest at 37 °C (Figure 3C). Enzyme activity is also affected by pH, so we measured the fluorescence intensity under different pH conditions. The results showed an ‘S’-shaped curve, with the fluorescence intensity increasing significantly between pH 7.0 and pH 8.0 (Figure 3D). We also studied the effects of the reaction time and AuMNP concentration. The results demonstrate that the fluorescence intensity gradually increased with time and plateaued at 60 min (Figure 3E). Additionally, the fluorescence intensity rapidly increased with an increasing AuMNP concentration up to 1.2 mg/mL, after which it tended to level off (Figure 3F). After comprehensive consideration, we selected a crRNA-Cas12a ratio of 1:2, ssDNA-FQ concentration of 400 nM (Figure S8), temperature of 37 °C, pH of 8.0, time of 60 min, and AuMNP concentration of 1.2 mg/mL for subsequent experiments.

### 3.6. The Sensitivity and Specificity of the Biosensor

**Figure 4:**
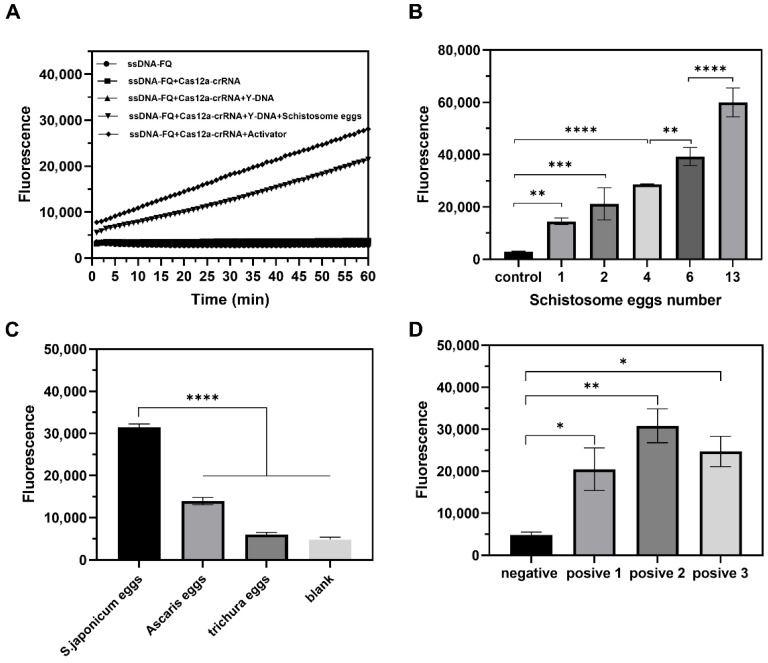
Magnetic-assisted Y-DNA-regulated CRISPR-Cas12a biosensor for detection of *S. japonicum* eggs. (**A**) The feasibility of detecting *S. japonicum* eggs. (**B**) Increase in fluorescence signal with number of eggs. (**C**) Selectivity of *S. japonicum* egg detection; the fluorescence intensity of *S. japonicum* eggs, Ascaris lumbricoides eggs, and Trichuris eggs. (**D**) Detection of eggs in rabbit fecal samples (*n* = 3; * *p* < 0.05, ** *p* < 0.01, *** *p* < 0.001, **** *p* < 0.0001).

The optimized reaction conditions were used to detect the egg mimic of *S. japonicum* (tDNA) and explore nucleic acid detection performance. Different concentrations of tDNA were added to the detection system, and it was found that the fluorescence intensity at 520 nm gradually increased with an increasing tDNA concentration (Figure S6A). Figure S6B shows a good linear relationship between the fluorescence intensity and target concentration in the range of 0.6–50 nM. The linear regression equation used was FL = 15835LgC + 100295, with an R^2^ of 0.9743. To test the specificity of the magnetic-assisted Y-DNA-regulated CRISPR-Cas12a biosensor for nucleic acid detection, dibase mismatch sequence (Mis-2), pentabase mismatch sequence (Mis-5), miR-21, piR-54265, miR-221-3p, and target tDNA were detected individually. The results in Figure S7 show that the fluorescence intensity of the base mismatch group was slightly lower than that of tDNA at the same concentration, while the fluorescence intensity decreased significantly when nucleic acid sequences with basically mismatched base sequences were added. This indicates that the detection platform was not sensitive to nucleic acid chains with several base mismatches but could sensitively identify basically mismatched sequences. In summary, the magnetic-assisted Y-DNA-regulated CRISPR-Cas12a biosensor demonstrated good performance in nucleic acid detection. We further investigated the ability of the magnetic-assisted Y-DNA-regulated CRISPR-Cas12a biosensor to detect non-nucleic acid substances by detecting *S. japonicum* eggs. Figure 4A shows that, in the presence of eggs, the activity of Cas12a-crRNA was activated, cutting ssDNA-FQ fluorescence reporter molecules and producing significant fluorescence signals. We also investigated the relationship between the number of *S. japonicum* eggs and the fluorescence intensity. The results in Figure 4B show that the fluorescence intensity of the detection system increased with an increase in the number of eggs, and the fluorescence intensity of the one-egg group was significantly higher than that of the control group. Furthermore, the fluorescence signal showed a strong linear correlation with the number of *S. japonicum* eggs (1–6 eggs, R^2^ = 0.9882; Figure S9), demonstrating that the assay can be reliably calibrated for quantitative analysis of egg burden. The specificity of the detection system was evaluated, and the results showed that the fluorescence intensity of the *S. japonicum* eggs was significantly higher than that of *Ascaris lumbricoides* and *Trichuris* eggs, confirming that the sensor had good specificity for targets (Figure 4C). In addition, we tested three infected rabbit fecal samples, and the results showed that the fluorescence intensity of positive samples was significantly higher than that of negative samples (Figure 4D). These results demonstrate that the magnetic-assisted Y-DNA-regulated CRISPR-Cas12a biosensor can be used to detect *S. japonicum* eggs with high sensitivity and specificity and that it has great potential for clinical diagnosis.

## 4. Conclusions

Overall, this study presents a magnetically assisted Y-DNA probe-regulated CRISPR-Cas12a biosensor platform for the highly sensitive detection of *S. japonicum* eggs. By integrating AuMNPs for nanoprobe synthesis and purification, the platform minimizes background noise and simplifies the detection workflow. The programmable Y-DNA aptamer probe enables precise target recognition and triggers Cas12a collateral cleavage, offering a versatile framework for detecting both nucleic acid and non-nucleic acid targets. Experimental results demonstrate exceptional sensitivity, distinguishing single eggs in complex biological samples and differentiating them from those of other parasitic species. This work contributes significantly to biosensor technology by combining magnetic-assisted sample preparation with CRISPR-based detection, providing a practical and scalable approach for clinical diagnostics and environmental monitoring. Compared to qPCR, our method offers several advantages: it is equally sensitive in detecting low numbers of *schistosomiasis* eggs, provides faster results with a shorter turnaround time, and is more cost-effective due to simpler reagent and equipment requirements. These benefits make it a promising alternative, especially in resource-limited settings. Future research should focus on enhancing the sensitivity, specificity, stability, and reproducibility of this biosensor platform, as well as expanding the range of detectable targets. With continued development, this biosensor platform holds great potential as a robust, adaptable tool for broad applications in parasitology and beyond.

## Data Availability

The data are available from the corresponding author upon reasonable request, subject to ethical and privacy restrictions.

## Supplementary Materials

The following supporting information can be downloaded at: https://www.mdpi.com/article/10.3390/bios16050293/s1, S1. Experimental Section details; Figure S1. (A) SEM image of AuNPs and (B) AFM image of AuNP; Figure S2. The UV absorption spectrum of the supernatant changed after AuNPs were modified on the surface of Fe_3_O_4_; Figure S3. The element mapping image of AuMNPs, (A) the HAADF-STEM (high-angle annular dark-field image–scanning transmission electron microscope) image of AuMNPs, (B) Fe element mapping image, (C) Au element mapping image, and (D) overlapping image of Fe and Au element mapping; Figure S4. The UV–vis quantification of Y-DNA on the surface of AuMNPs; Figure S5. Increase in the fluorescence signal of the biosensor over different concentrations of the activator; Figure S6. (A) Increase in the fluorescence signal of the sensor over different concentrations of tDNA and (B) quantitative tDNA detection; Figure S7. Selectivity of the biosensor for tDNA detection. The concentrations of tDNA, Mis-2, Mis-5, miR-21, piR-54265, and miR-221-3p were all 20 nM. Figure S8. Optimization of ssDNA-FQ concentration; Figure S9 Standard curve of fluorescence intensity against Schistosome egg count (1–6 eggs). Linear fit: y = 4779.66x + 9966.10, R^2^ =0.9882. Data are mean ± SD (n = 3) Table S1: Sequences of oligonucleotides used in the biosensor. References [30,32] are cited in the Supplementary Materials.
