# Supplementary material for: A Magnetic-Assisted CRISPR-Cas12a Biosensor Incorporating a Y-DNA Probe for Sensitive Detection of Schistosoma japonicum Eggs
Source: Biosensors (Basel). 2026 May 18;16(5):293. doi: 10.3390/bios16050293 (PMC13204850; doi:10.3390/bios16050293)
Supplement: Supplementary file 1 [file biosensors-16-00293-s001.zip › biosensors-4275175-supplementary.pdf]

Supporting Information

Article

A Magnetic-Assisted CRISPR-Cas12a Biosensor Incorporating a Y-DNA Probe for Sensitive Detection of *Schistosoma japonicum* Eggs

Ting Liu [1,†], Haogang Guo [2,†], Mengmeng Yu [1], Jiawei Peng [1], Liwen Guan [1], Shuying Xie [3], Xian Hao [1,*] and Yifei Yang [1,*]

[1] Jiangxi Provincial Key Laboratory of Preventive Medicine, School of Public Health, Nanchang University, Nanchang 330031, China; liutingo20@163.com (T.L.); yumengmengjx@163.com (M.Y.); 13627968672@163.com (J.P.); guanliwen116@163.com (L.G.)
[2] School of Queen Mary, Nanchang University, Nanchang 330031, China; guohaogang2004@163.com
[3] Jiangxi Provincial Institute of Parasitic Diseases, Nanchang 330031, China; xsy0317@163.com
* Correspondence: xian.hao@ncu.edu.cn (X.H.); yangyifei@ncu.edu.cn (Y.Y.)
† These authors contributed equally to this work.

S1. Experimental section detail

S1.1 Materials and reagents

The sequences of oligonucleotide in this strategy are shown in Table S1. They were purchased from Shanghai Sangon Biological Engineering Technology & Services Co., Ltd. (Shanghai, China). Trisodium citrate ($Na_3Ct\ 2H_2O$) and ethylene glycol (EG) were purchased from Chinese Pharmaceutical Chemical Reagent Co., Ltd. (China). Ferric trichloride ($FeCl_3\ 6H_2O$), chloroauric acid ($HAuCl_4$ 4H2O), sodium borohydride (NaBH4), sodium acetate (NaAc) and tris (2-carboxyethyl) phosphine hydrochloride (TCEP) were purchased from Aladdin (Shanghai, China). polyethyleneimine (PEI, MW25kDa) from McLean (China) and Engen ®LbaCas12a (cpf1) from NEBENGLAND (USA). *Schistosoma japonicum* eggs and other parasite eggs are provided by Jiangxi Institute of parasitic Diseases Control. Phosphate buffer (PBS, 10mM, pH=7.4) was prepared by standard method. The buffer solution was prepared with deionized water (resistivity ≥ 18.2 MΩ·cm). All other chemicals were used as received without further purification.

S1.2 Apparatus

Transmission electron microscope (TEM) images were run at 100kV on JEM-2100 transmission electron microscope (Japan). High resolution imaging and energy dispersion spectroscopy were performed at the same time. The range of X-ray diffraction on the X-ray diffractometer (Brook, Germany) is 20° to 80°. The UV-vis spectrum was recorded by UV-1800 spectrophotometer (Shimadzu, Kyoto, Japan). The fluorescence spectrum was recorded by RF-6000 fluorescence spectrophotometer

(Shimadzu, Kyoto, Japan). Real-time fluorescence quantitative PCR (Thermo Fisher Scientific Inc., Waltham, MA) was used to record the real-time fluorescence intensity.

S1.3 Synthesis of gold nanoparticles (AuNPs).

The preparation of AuNPs refers to the synthesis of 2-3 nm gold nanoparticles by reduction of chloroauric acid with sodium borohydride previously reported[1]. In short, 1 mL 1% HAuCl4 was added to 90 mL H_2O at room temperature (20-23 °C), stirring 1 min in a magnetic agitator, and then adding 2 mL 38.8 mM Na3Ct. After 1 min, the freshly prepared 1 mL 0.075 $NaBH_4$ was added to the solution and stirred for 5 min. Finally, the solution was kept away from light in a refrigerator at 4 °C.

S1.4 Synthesis of Fe_3O_4.

monodisperse magnetic Fe_3O_4 microspheres with a particle size of about 200 nm were synthesized by an improved solvothermal reaction [2]. In short, 1.08g $FeCl_3{\cdot}6H_2O$ and 0.25 g $Na_3Ct{\cdot}2H_2O$ dissolve in 20 mL Ethylene glycol (EG) to obtain mixed solution A. Then 2 g NaAc was added to another 20 mL EG solution and the mixed solution B was obtained by vibrating and dissolving. The mixed solution B is added to the mixed solution A under the condition of stirring to obtain the mixed solution C. The mixed solution C stirred 30 min violently, and then transferred the mixed solution C of 30 mL to a stainless steel autoclave with the capacity of 50 mL. The reactor was heated to 200 °C and kept at this temperature for 10 h, then cooled naturally to room temperature. Finally, the black precipitate was magnetically separated and washed with water and ethanol for 4 times, and the black sediment was dispersed in 50 mL ultra-pure water.

S1.5 Synthesis of AuNPs labeling Fe_3O_4 nanoparticles (AuMNPs).

AuNPs was modified on the surface of Fe_3O_4 microspheres by ultrasound to synthesize AuMNPs [2]. In short, 5 mL Fe_3O_4 suspension was dispersed in 50 mL 6 mg/mL PEI solution and treated with ultrasound for 1h. During this process, PEI was gradually self-assembled on the surface of Fe_3O_4 to form Fe_3O_4@PEI microspheres. Then the Fe_3O_4@PEI microspheres were magnetically separated and washed with ultra-pure water for 4 times to remove excess PEI. Then, Fe_3O_4@PEI microspheres were mixed with 2-3 nm AuNPs and treated with ultrasound for 30 min to form monodisperse AuMNPs microspheres. Finally, AuMNPs microspheres were magnetically separated and washed for 4 times, then dispersed in 40 mL ultra-pure water and stored at 4 °C. The final concentration of AuMNPs solution is about 0.4 mg/mL.

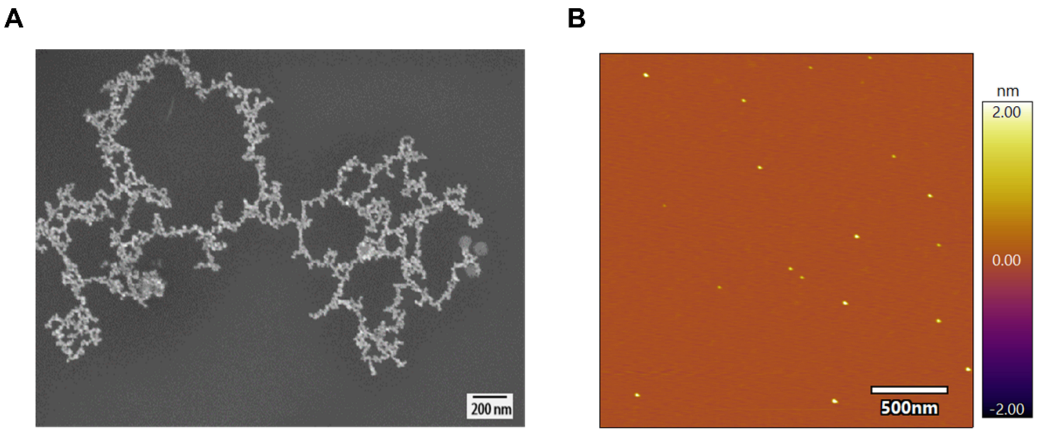

Figure S1. (A) SEM image of AuNPs and (B) AFM image of AuNPs.

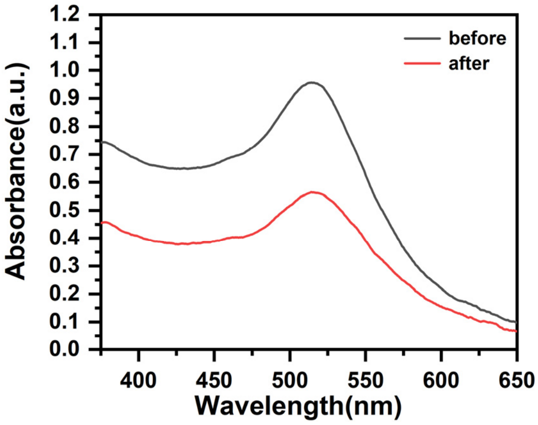

Figure S2. The UV absorption spectrum of the supernatant was changed after AuNPs was modified on the surface of Fe_3O_4. After a completion of reaction, the absorption intensity of the supernatant reduced by 40.89%.

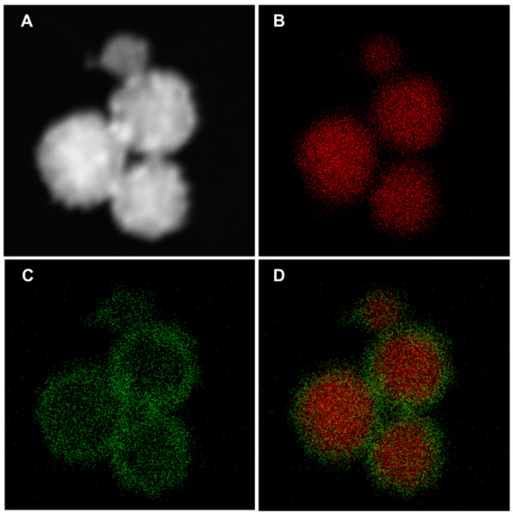

Figure S3. The element mapping image of AuMNPs. (A) the HAADF-STEM (high angle annular dark field image-scanning transmission electron microscope) image of AuMNPs. (B) Fe element mapping image. (C) the Au element mapping image. (D) the overlapping image of Fe and Au element mapping.

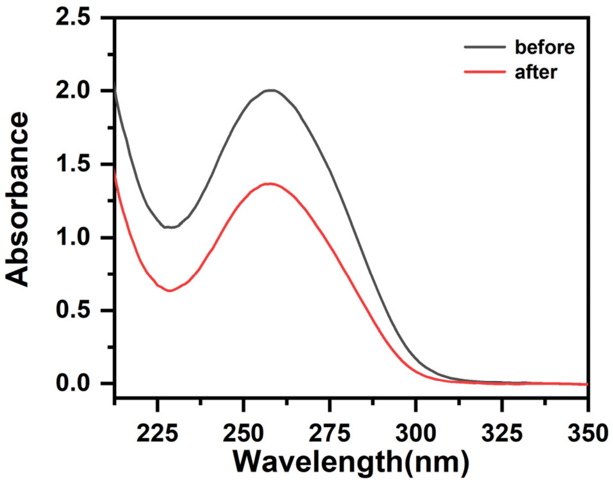

Figure S4. The UV-vis quantification of Y-DNA on the surface of AuMNPs. The quantity of Y-DNA probe on AuMNPs was calculated by the decrease of ssDNA concentration before and after reaction in supernatant.

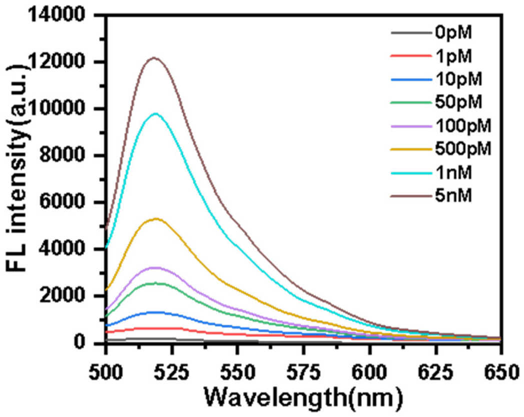

Figure S5. Fluorescence signal increase of the biosensor over different concentrations of activator.

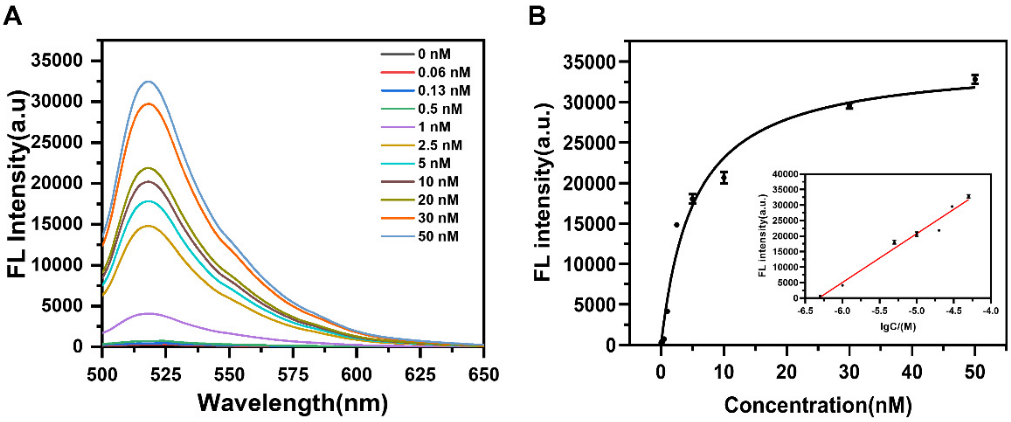

Figure S6. (A) Fluorescence signal increase of the sensor over different concentrations of tDNA. (B) Quantitative tDNA detection. Inset: linear relationship of different concentrations of tDNA.

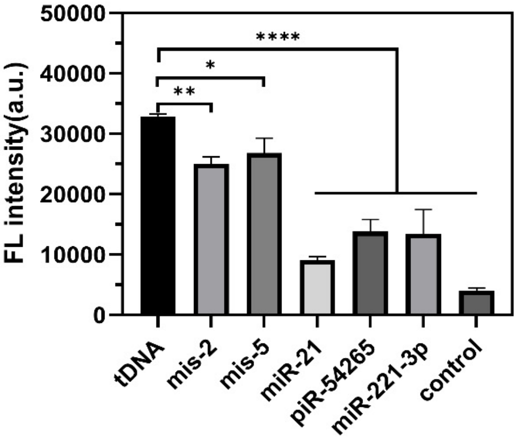

Figure S7. Selectivity of the biosensor for tDNA detection. The concentrations of tDNA, Mis-2, Mis-5, miR-21, piR-54265, and miR-221-3p were all 20 nM.

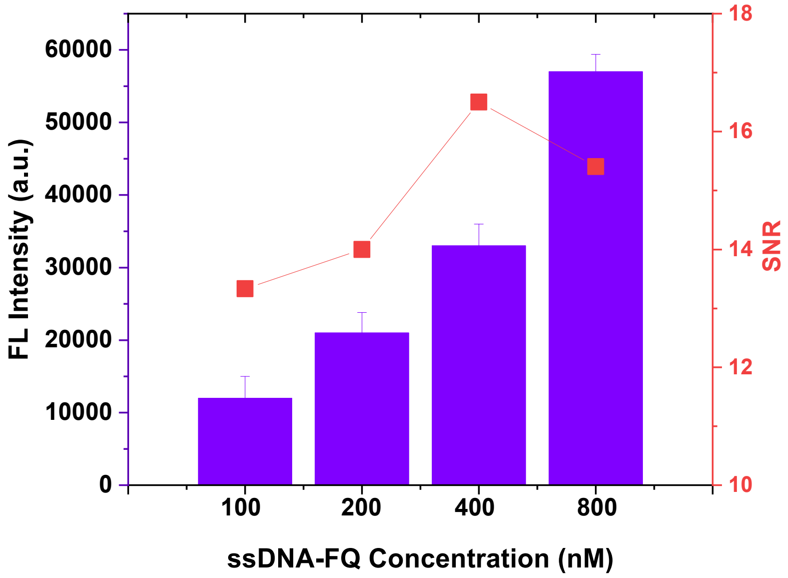

Figure S8. Optimization of ssDNA-FQ concentration.

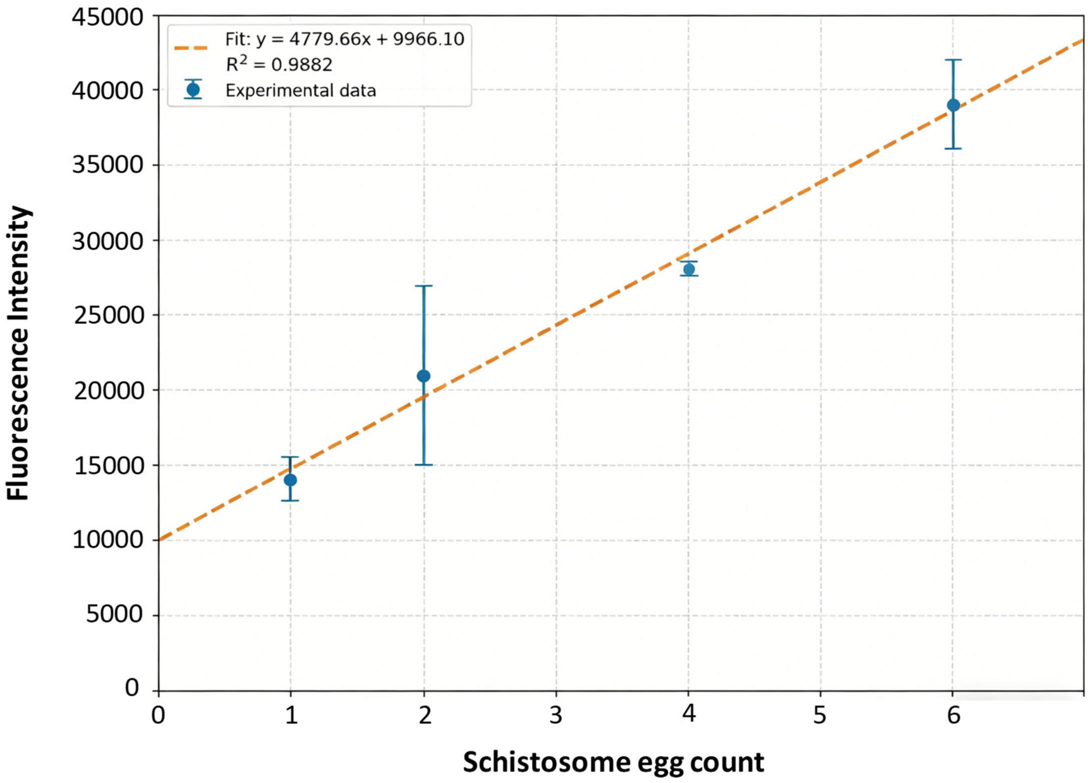

Figure S9. Standard curve of fluorescence intensity against Schistosome egg count (1–6 eggs). Linear fit: y = 4779.66x + 9966.10, R^2 =0.9882. Data are mean ±SD (n=3).

Table S1. Sequences of oligonucleotide used in this biosensor

name	Sequences（5' to 3'）
Captured DNA	TGAGATATAAAGGGCAGAAATAAGTAGGGG
Fix DNA	AAGAGCCCTACTTATTGTCACAG-SH
Activator	CTGTGACTCTGCCCTTTATTGA
ssDNA-FQ	6-FAM-TTATT-BHQ1
ssDNA	GAGCAGTCACAGTCCAGAAGGGCATGTCAGGGCTTGG ATACCTCGCATTCACCCTTGCACGATAC
Taget DNA	CCCCTACTTATTTCTGCCCTTTATATCTCA
Mis-2	CCCCTACTTATTTCTGCCGTTTATATCACA
Mis-5	CGCCTAGTTATTACTGCCGTTTATATCACA
miR-221-3p	AGC UAC AUU GUC UGC UGG GUU UC
piRNA-54265	UGGAGGUGAUGAACUGUCUGAGCCUGACC
miRNA-21	UAGCUUAUCAGACUGAUGUUGA
crRNA	UAAUUUCUACUAAGUGUAGAUUCAAUAAAGGGCAGA GUCAC

REFERENCES

[1] Brown K R, Walter D G, Natan M J. Seeding of colloidal Au nanoparticle solutions. 2. Improved control of particle size and shape [J]. Chem Mater, 2000, 12(2): 306-313.

[2] Wang C, Wang J, Li M, et al. A rapid SERS method for label-free bacteria detection using polyethylenimine-modified Au-coated magnetic microspheres and Au@Ag nanoparticles [J]. Analyst, 2016, 141(22): 6226-6238.
